# A Comparison of Changes in Anxiety and Depression Symptoms of Spontaneous Users and Trial Participants of a Cognitive Behavior Therapy Website

**DOI:** 10.2196/jmir.6.4.e46

**Published:** 2004-12-22

**Authors:** Helen Christensen, Kathleen M Griffiths, Ailsa E Korten, Kylie Brittliffe, Chloe Groves

**Affiliations:** ^1^The Centre for Mental Health ResearchThe Australian National UniversityCanberraAustralia

**Keywords:** Internet, mental health, depression

## Abstract

**Background:**

In randomized controlled trials Internet sites have been shown to be effective in the treatment of depression and anxiety. However, it is unclear if the positive effects demonstrated in these trials transfer to community users of such sites.

**Objective:**

To compare anxiety and depression outcomes for spontaneous visitors to a publicly accessible cognitive behavior therapy website (MoodGYM) (http://moodgym.anu.edu.au) with outcomes achieved through a randomized controlled efficacy trial of the same site.

**Methods:**

All community visitors to the MoodGYM site between April 2001 and September 2003 were sampled: 182 participants in the BlueMood Trial who had been randomly assigned to the MoodGYM site as part of a large trial and 19607 visitors (public registrants) to the site. Symptom assessments (quizzes) were repeated within the website intervention to allow the examination of change in symptoms across modules. Outcome variables were (1) age, gender, initial depression severity scores, and number of assessments attempted, and (2) symptom change measures based on Goldberg anxiety and depression scores recorded on a least two occasions.

**Results:**

Public registrants did not differ from trial participants in gender, age, or initial level of depression, which was high for both groups relative to previously published epidemiological data sets. Trial participants completed more assessments. No significant differences in anxiety or depression change scores were observed, with both public registrants and trial participants improving through the training program.

**Conclusions:**

Public registrants to a cognitive behavior therapy website show significant change in anxiety and depression symptoms. The extent of change does not differ from that exhibited by participants enrolled on the website for a randomized controlled trial.

## Introduction

The Internet is increasingly seen as a resource to disseminate self-help and clinician-based mental health interventions. To date, there have been 6 published trials evaluating the delivery of cognitive behavior therapy (CBT) using the Internet for anxiety and depression [1-6], of which 4 reported positive outcomes [1,2,4,5]. As yet, however, there has been no assessment of whether these evaluated websites are as useful for public users as for trial participants. The experience of a user of an open website is less structured than the experience of a participant in a randomized controlled trial (RCT). For example, RCT participants are encouraged by trial managers, either in person or by phone. They are tracked and monitored over the trial period, and may be called to complete questionnaires over the phone or on the Internet. These aspects of the intervention may be responsible for the efficacy of these sites, either directly through personal support provided to the participant, or indirectly through the greater adherence that results from this support.

In this paper, we compare the mental health outcomes of public registrants and trial participants using the MoodGYM website, a cognitive behaviour therapy website [1, 7]. Public registrants directed themselves to the site using search engines and links from relevant Web pages. Trial participants were Internet users in the community in Canberra, Australia, who had elevated depression symptoms. Previous research on the site has demonstrated the effectiveness of the site in an RCT (subsequently referred to as the "BlueMood Trial") [1] and change in symptoms by site users [7]. However, no previous investigation has directly compared treatment outcomes from the two samples, or evaluated factors of age or initial depression severity in treatment outcome.

## Methods

### Sample

All participants, except 71 students (who were recruited via tutorial) were recruited from among MoodGym registrants between April 2001 and September 2003 [7]. Registrants are individuals who enroll on the site (by providing a name and password) and who create a record in our database. The BlueMood trial participants were recruited by survey and subsequently randomized to the MoodGYM website as part of a three arm trial (the other arms were a psycho-education intervention and an attention placebo condition; see [1]). Trial participants scored more than 12 on the Kessler Psychological Distress Scale [8] and were not receiving clinical care from either a psychologist or a psychiatrist at the time of recruitment. The latter requirement was imposed so that the trial included individuals who were the target audience for self-help interventions and excluded individuals who might already be receiving CBT. Because in this paper our central goal was to evaluate change in symptom scores over the course of the MoodGYM training program, only those individuals from both samples who completed more than one assessment on the website were included.

There were 19789 people registered between April 2001 and September 2003, of whom 182 were participants in the BlueMood RCT trial, and 19607 were members of the public who had registered online. Among the public registrants, 12141 (61.9%) completed at least one quiz, but only 3055 (15.6%) completed at least 2 of the Goldberg Depression Scales. Among the BlueMood RCT trial participants, 157 (86.3%) completed at least one quiz, while 121 (66.5%) completed 2 or more of the Goldberg depression assessments.

**Table 1:** Mean (SD) and n of the Goldberg Depression and Goldberg Anxiety Scales for the first module

		**Number of modules completed**	**Public Registrants**		**BlueMood Trial Participants**		**Total**	
			**Mean (SD)**	**n**	**Mean (SD)**	**n**	**Mean (SD)**	**n**
**Depression Scale**								
	**Male**	**1**	5.23 (2.51)	2266	6.50 (1.29)	4	5.23 (2.51)	2270
		**2 or more**	5.37 (2.56)	782	6.07 (1.86)	27	5.40 (2.54)	809
		**Total**	5.27 (2.52)	3048	6.13 (1.78)	31	5.28 (2.52)	3079
	**Female**	**1**	5.37 (2.38)	4983	4.79 (1.93)	14	5.37 (2.38)	4997
		**2 or more**	5.70 (2.32)	1842	5.55 (2.02)	86	5.69 (2.31)	1928
		**Total**	5.46 (2.37)	6825	5.44 (2.01)	100	5.46 (2.36)	6925
	**Total**	**1**	5.33 (2.42)	7249	5.17 (1.92)	18	5.33 (2.42)	7267
		**2 or more**	5.60 (2.40)	2624	5.67 (1.98)	113	5.60 (2.38)	2737
		**Total**	5.40 (2.42)	9873	5.60 (1.98)	131	5.40 (2.41)	10004
**Anxiety Scale**								
	**Male**	**1**	5.22 (2.56)	2393	5.60 (1.52)	5	5.22 (2.56)	2398
		**2 or more**	5.41 (2.61)	537	5.37 (2.51)	30	5.41 (2.60 )	567
		**Total**	5.25 (2.57)	2930	5.40 (2.38)	35	5.25 (2.57)	2965
	**Female**	**1**	5.74 (2.47)	4932	5.38 (1.47)	24	5.74 (2.46)	4956
		**2 or more**	5.93 (2.41)	1014	5.83 (2.13)	87	5.92 (2.39)	1101
		**Total**	5.77 (2.46)	5946	5.73 (2.00)	111	5.77 (2.45)	6057
	**Total**	**1**	5.57 (2.51)	7325	5.41 (1.45)	29	5.57 (2.51)	7354
		**2 or more**	5.75 (2.49)	1551	5.71 (2.23)	117	5.75 (2.47)	1668
		**Total**	5.60 (2.51)	8876	5.65 (2.10)	146	5.60 (2.50)	9022

That is, 3176 participants provided sufficient data to allow change in depression scores to be assessed (see Table 1). Change in Goldberg anxiety score could be assessed for 1668 people, all of whom had completed 2 or more anxiety scales. Appendix A provides exact participant numbers for the major analyses.

### Site Description

The site consists of a set of 5 cognitive behavioral training modules, a personal workbook (containing 29 exercises and assessments) that records and updates each user's responses), an interactive game, and a feedback evaluation form. Module 1 introduces the site “characters” (who model patterns of dysfunctional thinking) and demonstrates the way mood is influenced by thinking, using animated diagrams and interactive exercises. Module 2 describes types of dysfunctional thinking and the methods to overcome them, and provides scales for self-assessment of “warpy” (dysfunctional) thoughts. Module 3 provides behavioral methods to overcome dysfunctional thinking, and includes sections on assertiveness and self-esteem training. Module 4 assesses life-event stress, pleasant events, and activities, and provides three downloadable relaxation tapes. Module 5 covers simple problem-solving and typical responses to relationship break-up. Workbook exercises are integrated into each of the modules.

Each module was designed to take between 30 and 45 minutes to complete, but users can opt to skip sections. Module 1 has approximately 30 pages but many of these contain browser-supported interactive features (creating additional pages) and supplementary pop-up windows. Module 3 has more than 60 pages, but users are directed to specific sections depending on their scores on earlier tests and thus may not access all pages (see multimedia presentation [7]). Although users were encouraged to proceed through the assessments and modules in order, they were free to move about within the site at will. Thus, some registrants started with later modules and did not necessarily work through them in order; however, research previously conducted that matched log files to user profiles indicated that most individuals do approach the modules sequentially [7]. No assessments were compulsory in the original MoodGYM site. Since September 2003, however, a new version of the software has been installed (Mark II) that consists of compulsory core assessments and a requirement that modules be completed in order.

The MoodGYM site provides CBT for depression over 5 modules. Users complete online anxiety and depression scales in each of the 5 modules. This allows us to assess whether anxiety and depression scores change over the training program.

### Assessments

Online assessments included the anxiety and depression items from the Goldberg Depression and Anxiety Scales [9]. Each of the Goldberg scales comprises 9 items. These assessments were introduced at the beginning of each of the modules, so it was possible for individuals to complete between 1 and 5 assessments. Other online assessments included the Warpy Thoughts Quiz, a 42-item scale that measured dysfunctional thinking [10]; the Life Whacks Questionnaire (adapted with permission from the Tennant & Andrews scale to measure the stress of life events) [11]; the Measure of Parenting Style (the MOPS, used with permission from G. Parker) [12]; and the Pleasant Events Schedule [13]. These additional assessments are not reported on in this paper. Age and gender variables were collected at registration, and initial level of depression was measured by Goldberg scales at the beginning of module 1.

### Analysis Strategy

Differences in response rates were analyzed using logistic regression. Differences in symptom change scores were analyzed using analysis of variance (ANOVA) with repeated measures using SPSS software. Models predicting final depression score accounting for initial score were derived from linear regression models. SPSS 11.5 software was used [14].

## Results

### Demographic Variables

Two thirds of all registrants were female (66.1%). Among those registered, females were more likely than males to complete at least 2 Goldberg Depression Scales (odds ratio [OR] controlling for sample [public or trial] 1.24; 95% confidence interval [CI] 1.14-1.34), while there was no difference between the sexes for completion of at least two anxiety scales (OR 0.97; 95% CI 0.87-1.1). Appendix A provides full participant numbers.

### Initial Depression and Anxiety Scores

Initial levels of depression, as measured by the Goldberg Depression Scale in module 1, are shown in Table 1. Differences between the samples were not significant (F[1, 10004]=1.25; *P*=.26), nor were any interactions between gender and number of modules completed (F[1, 10004]=2.661; *P*=.10) Those who completed more than one module had significantly higher scores (F[1, 10004]=15.7; *P*<.001). Appendix B provides full details of analysis.

Initial levels of anxiety are shown in Table 1. Differences between the samples were not significant, nor were any interactions between sample, gender, or number of modules completed (see Appendix B).

### Change in Depression and Anxiety Scores

For 3176 people, it was possible to compare the initial and final completed depression scales, although there were only 138 for whom the profile across all 5 modules could be traced. The change in depression score between the first and last modules attempted was analyzed using repeated measures ANOVA controlling for sample, gender, number of modules completed (1 to 5), and all interaction terms. The sample numbers are presented in Appendix C. Sample (public or trial) was not significant, nor were any of the interaction terms. A final model included gender and number of modules completed; the parameter estimates are shown in Table 2.

**Figure 1:**
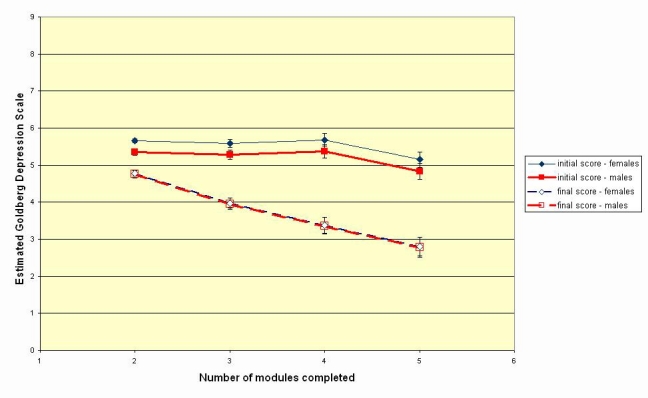
Estimated means with standard error bars for initial and final scores on Goldberg Depression Scale, by gender and number of modules completed, as derived from repeated measures ANOVA with fixed factors being gender and number of modules completed

Improvement increased as the number of modules completed increased from 2 to 4, but there was no significant difference in estimated improvement between 4 and 5 modules completed. Females had higher depression scores than males for the first module, but there were no differences between the sexes for the final depression score. Figure 1 shows the estimated marginal means for the first and final modules completed, by gender and number of modules completed.

**Figure 2:**
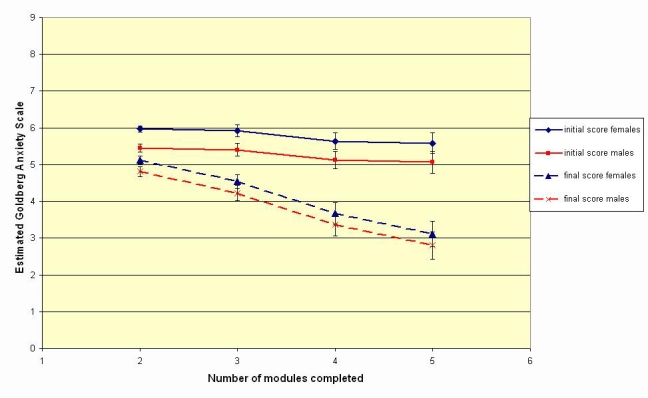
Estimated means with standard error bars for initial and final scores on Goldberg Anxiety Scale, by gender and number of modules completed, as derived from repeated measures ANOVA with fixed factors being gender and number of modules completed

For 1668 people, it was possible to compare the first and last completed anxiety scales, although there were only 76 for whom the profile across all 5 modules could be traced. The change in anxiety score between the initial and final modules attempted were analyzed using repeated measures ANOVA controlling for sample (public or trial), gender, number of modules completed (1 to 5) and all interaction terms. Neither sample type nor any of the interaction terms was significant.

A final model included gender and number of modules completed; parameter estimates are shown in Table 2. Improvement increased as the number of modules completed increased from 2 to 4, but there was no significant difference in estimated improvement between 4 or 5 modules completed. Females had higher scores than males. Figure 2 shows the estimated marginal means for the first and final modules completed, by gender and number of modules completed.

**Table 2:** Parameter estimates for prediction of first and last Goldberg depression and Goldberg anxiety scores completed as derived from repeated measures ANOVA with factors being gender and number of modules completed

	**Dependent Variable**	**Parameter**	**B**	**S.E.**	**t**	***P*-value**
**Depression**						
	**Initial depression score**	Intercept	5.15	0.21	24.83	<.001
		Male	-0.32	0.09	-3.39	<.001
		2 modules completed	0.52	0.21	2.43	.02
		3 modules completed	0.45	0.23	1.92	.05
		4 modules completed	0.53	0.27	1.99	.05
		5 modules completed *	0.00	.	.	.
	**Final depression score**	Intercept	2.80	0.26	10.95	<.001
		Male	-0.02	0.12	-0.16	.87
		2 modules completed	1.98	0.26	7.57	<.001
		3 modules completed	1.18	0.29	4.13	<.001
		4 modules completed	0.58	0.33	1.76	.08
		5 modules completed *	0.00	.	.	.
**Anxiety**						
	**Initial anxiety score**	Intercept	5.58	0.28	19.65	<.001
		Male	-0.52	0.13	-4.05	<.001
		2 modules completed	0.39	0.29	1.33	.18
		3 modules completed	0.34	0.32	1.06	.29
		4 modules completed	0.06	0.36	0.16	.88
		5 modules completed *	0.00	.	.	.
	**Final anxiety score**	Intercept	3.11	0.35	8.94	<.001
		Male	-0.31	0.16	-2.01	.04
		2 modules completed	2.01	0.36	5.63	<.001
		3 modules completed	1.43	0.39	3.64	<.001
		4 modules completed	0.56	0.45	1.26	.21
		5 modules completed *	0.00	.	.	.

^*^ excluded category

### Predicting Depression Scores at End of the Intervention

**Figure 3:**
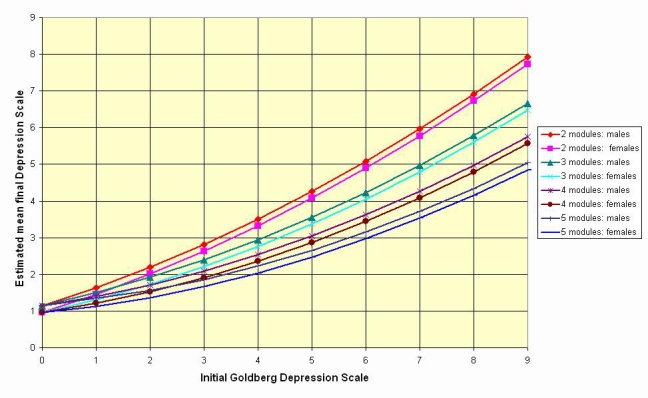
Estimated final Goldberg depression score by initial score, number of modules completed, and gender. Derived from general linear model with final score as dependent variables, gender, and number of modules completed as fixed factors, and linear and quadratic terms for the Goldberg scale as covariates

We also examined the expected magnitude of the final score for different starting levels of depression. In a linear regression with dependent variable equal to the final score and independent variables gender, number of modules (treated as 3 dummy variables), initial score, and a quadratic term in the initial score, all independent variables and the interaction between initial score and number of modules were significant. Males are expected to be 0.193 (SE = .095) higher than females, controlling for the initial level and number of modules. For initial scores above 2, it is expected that the final score will indicate improvement, the improvement increasing with the number of modules attempted. Figure 3 illustrates these relationships for the Goldberg Depression Scale.

## Discussion

The present study evaluated the outcomes of public registrants using MoodGYM delivered openly on a website by comparing them with trial participants in a RCT using the same site. We found that there were no differences in the initial level of depression or anxiety in the samples, that the gender balance did not differ significantly, and that the rate of change in symptoms was not different for the two samples. Both public registrants and trial participants exhibited significant improvement in symptoms.

Our findings suggest that MoodGYM can be used effectively in the community. If we had found stronger effects in the BlueMood Trial participants, we might have concluded that structured weekly human contact was a necessary condition for the site to deliver effective outcomes. However, we conclude that community users of the site are similar in gender distribution and severity of depression, and that site exposure results in similar significant symptom improvement.

Although there were no differences in outcome among participants who stayed in the program and completed at least 2 modules, we did find that community users were less likely than the trial participants to adhere to the full treatment program. Thus, the community users infrequently progressed beyond the first module of the program. Among the public, only 15.6% completed 2 or more of the modules, while over 66% of the trial participants completed 2 or more of the modules. This finding suggests that the formal structure of the trial may be important for compliance. Merrill et al reported a similar finding for clinic-based CBT: community clients attended substantially fewer sessions than clients in RCTs, yet “still showed similar levels of improvement” [15]. Only 15% completed 2 or more modules, a finding that may reflect the usability of the site, the acceptability of CBT type interventions, commitment to change, symptom level, user preference, or other factors. Factors such as these are likely to influence the uptake of non-Internet-based services as well, although comparative published data on potential users of standard health care services are not readily available. The low completion rate is not a major problem for free Internet services, which do not have specific costs based on user numbers (few additional costs are incurred for large numbers of non-completers).

There are limitations to the present study that need to be acknowledged. As is the case for studies using a benchmarking strategy, the samples involved are likely to be quite different given the operation of selection biases. The trial participants were Canberra-based, and from a population sample with high education and occupation levels. In contrast, the user sample is international, with participants from more than 62 countries. There was selective attrition, with many participants from the public sample dropping out before completion, and greater retention for the trial sample (although there were many opportunities for trial participants to be excluded or to drop out before randomization). These sample differences are difficult to characterize. However, they should be acknowledged as having the potential to mask differences in outcomes. Other sample characteristics, such as the concurrent use of evidence-based treatments other than psychotherapy (antidepressants, other medications, physical activity), were not measured but did not preclude participation in either sample.

The possibility cannot be ruled out that community users were assisted in the program by clinicians or other counselors. If this occurred for the majority of community users, our claim that the trial participants were the subject of greater assistance/support would be invalid. However, we consider it highly unlikely that the majority of public participants were assisted by a counselor or other person.

Clinicians and researchers have argued that although treatment *efficacy* needs to be established, it is crucial to demonstration of the *effectiveness* of treatments in real world settings [15]. Demonstrations of real-world effectiveness often employ benchmarking strategies where RCTs are chosen to compare results to community settings [16]. We have employed this strategy in the present study using outcomes from our own RCT. Unlike benchmarking in clinical settings where there can be considerable flexibility in application of the clinical therapy, Internet sites have the advantage of transferring the treatment with fidelity, so that differences that may exist between the trial and the real-world site can be more reliably attributed to external factors such as degree of human contact, capacity to maintain compliance, sample characteristics, and intensity of monitoring.

The findings of the present study demonstrate the effectiveness of Internet-based interventions in the early treatment and prevention of depression. Health systems in developed countries are expected to change radically over the next 10 years, with self-help and self-responsibility for health forming a new tier of the health system [17]. Sites such as MoodGYM are likely to provide both tools for the self-delivery of evidence-based prevention/treatment and resources to be used as adjuncts to professionally managed primary care.

## Appendix A

**Table 3:** Participant numbers for all analyses §

		**Public**	**BlueMood trial**	**Total**
**Total number registered**	**Males**	6640 (33.9%)	52 (28.6%)	6692 (33.8%)
	**Females**	12967 (66.1%)	130 (71.4%)	13097 (66.2%)
	**Total**	19607 (100%)	182 (100%)	19789 (100%)
**Completed at least one quiz**	**Males**	3823 (31.5%)	40 (25.5%)	3863 (31.4%)
	**Females**	8318 (68.5%)	117 (74.5%)	8435 (68.6%)
	**Total**	12141 (100%)	157 (100%)	12298 (100%)
**Completed 2 or more Goldberg Depression Scales***	**Males**	918 (30.0%)	30 (24.8%)	948 (29.8%)
	**Females**	2137 (69.9%)	91 (75.2%)	2228 (70.1%)
	**Total**	3055 (100%)	121 (100%)	3176 (100%)
**Completed 2 or more Goldberg Anxiety Scales**	**Males**	537 (34.6%)	30 (25.6%)	567 (34.0%)
	**Females**	1014 (65.4%)	87 (74.4%)	1101 (66.0%)
	**Total**	1551 (100%)	117 (100%)	1668 (100%)
**Completed both Warpy Thoughts quizzes**	**Males**	34 (34.7%)	10 (25.0%)	44 (31.9%)
	**Females**	64 (65.3%)	30 (75.0%)	94 (68.1%)
	**Total**	98 (100%)	40 (100%)	138 (100%)
**Completed Life Whacks quiz**	**Males**	268 (36.2%)	20 (20.6%)	288 (34.4%)
	**Females**	473 (63.8%)	77 (79.4%)	550 (65.6%)
	**Total**	741 (100%)	97 (100%)	838 (100%)
**Completed Mum and Dad quiz**	**Males**	107 (39.2%)	27 (26.5%)	134 (35.7%)
	**Females**	166 (60.8%)	75 (73.5%)	241 (64.3%)
	**Total**	273 (100%)	102 (100%)	375 (100%)
**Completed Pleasant Events quiz**	**Males**	103 (37.2%)	26 (24.8%)	129 (33.8%)
	**Females**	174 (62.8%)	79 (75.2%)	253 (66.2%)
	**Total**	277 (100%)	105 (100%)	382 (100%)

^*^ Among participants who completed at least 1 quiz, all but 125 completed at least 1 Goldberg Depression Scale.

^§^ 1370 people completed the quizzes for module 2 and 566 completed them for module 5.

## Appendix B

Linear regression model with initial levels of depression and anxiety as independent variables for participants who completed at least 2 modules

**Table 4:** Tests of between-subject effects (depression)

**Source**	**Type III Sum of Squares**	**df**	**Mean Square**	**F**	**P**
**Corrected model**	255.616	5	51.123	8.817	<.001
**Intercept**	11555.687	1	11555.687	1993.061	<.001
**Gender**	6.402	1	6.402	1.104	.29
**Module number***	91.030	1	91.030	15.700	<.001
**Sample**	7.265	1	7.265	1.253	.26
**Gender x****module number**	15.431	1	15.431	2.661	.10
**Gender x sample**	22.536	1	22.536	3.887	.049
**Error**	57968.006	9998	5.798		

^*^ Module number refers to whether participants had at least 2 completed tests for either the depression or anxiety tests.

**Table 5:** Tests of between-subject effects (anxiety)

**Source**	**Type III Sum of Squares**	**df**	**Mean Square**	**F**	**P**
**Corrected model**	582.699	7	83.243	13.435	<.001
**Intercept**	6817.017	1	6817.017	1100.211	<.001
**Gender**	5.640	1	5.640	.910	.34
**Sample**	5.084E-02	1	5.084E-02	.008	.93
**Module number**	1.259	1	1.259	.203	.65
**Gender x sample**	2.246	1	2.246	.362	.55
**Gender x module number**	1.629	1	1.629	.263	.61
**Sample x module number**	9.467E-02	1	9.467E-02	.015	.90
**Error**	55851.618	9014	6.196		
**Total**	339306.000	9022			
**Corrected Total**	**56434.317**	**9021**			

## Appendix C

**Table 6:** Number of people who completed at least 2 modules by the total number of depression modules completed

	**Number of Modules Completed**	**Public Registrants** **n**	**BlueMood Trial Participants** **n**	**Total** **n**
				
**Males**	2	677	5	682
	3	152	6	158
	4	60	12	72
	5	29	7	36
	at least 2	918	30	948
				
**Females**	2	1611	18	1629
	3	347	23	370
	4	111	16	127
	5	68	34	102
	at least 2	2137	91	2228
				
**Total**	2	2288	23	2311
	3	499	29	528
	4	171	28	199
	5	97	41	138
	at least 2	3055	121	3176

## Abbreviations

CBT: Cognitive behavior therapy
RCT: Randomized controlled trial
